# Updating the modified Thompson test by using whole-body bioluminescence imaging to replace traditional efficacy testing in experimental models of murine malaria

**DOI:** 10.1186/s12936-019-2661-x

**Published:** 2019-02-15

**Authors:** Diana Caridha, Mark Hickman, Lisa Xie, Franklyn Ngundam, Erin Milner, Amanda Schenk, Kirk Butler, Dylan Nugent, Patricia Lee, Norma Roncal, Susan Leed, Eve Hosford, Jangwoo Lee, Richard J. Sciotti, Gregory Reichard, Chad Black, Mara Kreishman-Deitrick, Qigui Li, Brian Vesely

**Affiliations:** 1 Walter Reed Army Institute of Research, 503 Robert Grant Avenue, Silver Spring, MD 20910 USA; 2 Biodefense Therapeutics, JPdM-BDTx, 110 Thomas Johnson Dr., Frederick, MD USA; 3 Dwight David Eisenhower Army Medical Center, 300 E Hospital Rd, Ft Gordon, GA 30905 USA; 4 National Institutes of Health, Office of Biodefense, Research Resources and Translational Research, 5601 Fishers Lane, Bethesda, MD 20892 USA

**Keywords:** *Plasmodium berghei*, Animal models, Refinement, Parasite load predicted parasitaemia (PLPP), Anti-malarial drugs, Preemptive euthanasia, Bioluminescence, Parasitaemia readout, In vivo imaging technology

## Abstract

**Background:**

Rodent malaria models are extensively used to predict treatment outcomes in human infections. There is a constant need to improve and refine these models by innovating ways to apply new scientific findings and cutting edge technologies. In addition, and in accordance with the three R’s of animal use in research, in vivo studies should be constantly refined to avoid unnecessary pain and distress to the experimental animals by using preemptive euthanasia as soon as the main scientific study objective has been accomplished.

**Methods:**

The new methodology described in this manuscript uses the whole-body bioluminescence signal emitted by transgenic, luciferase-expressing *Plasmodium berghei* parasites to assess the parasite load predicted parasitaemia (PLPP) in drug and control treated female ICR-CD1 mice infected with 1 × 10^5^ luciferase-expressing *P. berghei* (ANKA strain) infected erythrocytes. This methodology can replace other time-consuming and expensive methods that are routinely used to measure parasitaemia in infected animals, such as Giemsa-stained thin blood smears and flow cytometry.

**Results:**

There is a good correlation between whole-body bioluminescence signal and parasitaemia measured using Giemsa-stained thin blood smears and flow cytometry respectively in donor and study mice in the modified Thompson test. The algebraic formulas which represent these correlations can be successfully used to assess PLPP in donor and study mice. In addition, the new methodology can pinpoint sick animals 2–8 days before they would have been otherwise diagnosed based on behavioural or any other signs of malaria disease.

**Conclusions:**

The new method for predicting parasitaemia in the modified Thompson test is simple, precise, objective, and minimizes false positive results that can lead to the premature removal of animals from study. Furthermore, from the animal welfare perspective of replace, reduce, and refine, this new method facilitates early removal of sick animals from study as soon as the study objective has been achieved, in many cases well before the clinical signs of disease are present.

**Electronic supplementary material:**

The online version of this article (10.1186/s12936-019-2661-x) contains supplementary material, which is available to authorized users.

## Background

Despite reduction in malaria morbidity and mortality over the last 20 years, the World Health Organization has estimated that there were 212 million cases of malaria with 429,000 deaths in 2015, and 92% of those in Africa (World Health Organization, Fact Sheet: World Malaria Report 2016 WHO, 2016). While progress in treatment of malaria has reduced the burden of disease, resistance to all classes of available anti-malarial drugs, especially the artemisinin class of compounds, threatens the progress that has been made in patient treatment and increases the economic costs from disease in these already impoverished countries [1–5]. The fight for eradicating uncomplicated and severe malaria is far from over, and drug discovery and development of anti-malarial drugs is as important as ever [6].

Rodent malaria models, are extensively used to predict treatment outcomes in human infections. Four *Plasmodium* species (*Plasmodium chabaudi*, *Plasmodium vinckei*, *Plasmodium berghei*, and *Plasmodium yoelii*), which display different parasite biology and pathogenicity, as well as multiple mouse species, such as C57BL/6, CBA/T6, BALB/c, ICR-CD1, DBA/2J, are used in these animal models of malaria drug discovery such as the modified Thompson test of malaria cure and the Peters’ 4-day suppressive test. Among those, the ICR-CD1/*P. berghei* model is considered to be a good representative of the severe malaria disease in rodents [7]. These assays are used as primary and/or secondary screens before lead compounds are tested in non-human primates (NHPs) and remain a standard part of the malaria drug discovery and development process [7–12]. Many of the anti-malarial drugs currently in use were developed using the *P. berghei* murine models [12, 13]. Despite the ongoing discussion within the scientific community about whether the mouse model using *P. berghei* ANKA strain is an acceptable model for complicated human malaria disease, which includes cerebral malaria caused by *Plasmodium falciparum*, it is clear that there are many similarities between some rodent models and uncomplicated and cerebral malaria which makes these models indispensable and proven tools for anti-malarial drug discovery [8, 9, 13–16]. There is a constant need to improve rodent malaria models by innovating ways to apply new scientific findings and cutting edge technologies. Furthermore, in vivo models of infectious disease that involve progression to animal death have a need to establish at the earliest point in time when the main scientific objective of a study has been accomplished [17]. A “science-driven approach for termination of animal studies in chronic infection” strives to continuously refine animal models to minimize pain and suffering is a necessity [17]. In accordance with the three R’s (Replacement, Reduction, and Refinement) [18] of animal testing, all in vivo studies should be designed to avoid unnecessary pain, distress, and suffering to the experimental animals [19–22]. Modification of experimental design and study endpoint(s) to enhance the quality of scientific data gathered and improve animal welfare is a very important aspect of the refinement process [20]. In addition, the ability to predict animal death with accuracy and the use of preemptive euthanasia before experimental animals reach a moribund state is of paramount importance. If used correctly, preemptive euthanasia would not only significantly reduce unnecessary terminal pain and distress in experimental animals, but would also reduce study costs and increase refinement by making it possible to collect specimens and study data that otherwise would be lost [20, 21].

Accurate parasite quantitation in experimental rodent malaria is extremely important as the reduction in blood parasitaemia usually serves as the main experimental endpoint in these models. Microscopy, genetic-probe assays, polymerase chain reaction (PCR), nucleic acid-based probes, and transgenic *Plasmodium* spp. parasites are some of the most common techniques that are successfully employed to quantitate circulating parasites. Use of light-microscopy Giemsa-stained blood smears is the gold standard for detecting, identifying, and quantitating malaria parasites in the clinic and the experimental malaria studies [23–25]. This method is sensitive and relatively inexpensive, but it is labour-intensive and time-consuming. Most importantly, the accuracy of the Giemsa-stained thin blood smears readings greatly depends on interpretation of blood smears by technically competent staff [25]. Quantitative PCR and RT-PCR measurements are very sensitive methods for quantitating parasitaemia but these procedures are long and expensive to conduct [25–27]. Furthermore, PCR is an invasive procedure which cannot be used to follow the evolution of parasite burden associated with extended longitudinal in vivo studies. Like PCR, the use of flow cytometry protocols for quantitating malaria parasites through the use of nucleic acid staining dyes provides an accurate, reliable, and high throughput method [28–35]. However, flow cytometry methods involve the use of special dyes and complex protocols, and these techniques are not practical for rapid assessment of animal blood parasitaemia, such as quantitating the parasitaemia burden in donor animals to be used for the infection of animal colonies before in vivo drug discovery studies begin. Furthermore, flow cytometry methods cannot be used for rapid determination of blood parasitaemia before symptomatic animals are removed from in vivo drug discovery studies which may lead to false positive results and unnecessary removal of animals from studies. The development of transgenic luciferase-expressing *Plasmodium* parasites in the 1990s has had a significant impact on studying different aspects of malaria infection [36–38]. In particular, luciferase-expressing *P. berghei* parasites have been extensively used for in vitro and in vivo experimental malaria studies to quantitate parasitaemia in blood samples, monitor liver stage burden, assess drug efficacy during the liver and blood stages of disease, and to visualize parasite development throughout the *Plasmodium* life cycle [27, 36, 39–44]. Studies have shown that there is a strong correlation between quantitative assessments of malaria parasitaemia using flow cytometry and Giemsa-stained thin smear microscopy [28, 30, 31]. Miller et al. have shown that there is a good correlation between RT-PCR and bioluminescence image quantification of the parasite burden in livers of mice infected with luciferase-expressing *P. yoelii* parasites [26]. In addition, in vivo GFP and bioluminescence imaging of liver and blood stages of *P. berghei* allows for accurate quantitative measurement of parasite burden which is comparable to microscopy [32].

The modified Thompson test is utilized to assess the blood stage efficacy of potential anti-malarial compounds to eliminate parasitaemia resulting from a challenge infection of *P. berghei* parasites in female ICR-CD1 mice over a period of 31 days [7]. These tests are conducted bi-monthly in groups of 40 mice that include six groups of experimental compounds, and positive and negative control groups. Measuring the parasitaemia of donor and study animals before, during, and on the 31st day of the study is a labour intensive and costly process that lasts from several hours to several days and demands the involvement of highly qualified technical staff. The new method described in this manuscript uses the whole-body bioluminescence signal emitted by transgenic, luciferase-expressing *P. berghei* parasites to assess the parasite load predicted parasitaemia (PLPP) in drug and control treated malaria-infected female ICR-CD1 mice. Use of this new method has made it possible to totally eliminate the use of Giemsa-stained thin blood smear microscopy and has reduced the use of flow cytometry for quantifying blood parasitaemia to a minimum for confirmatory use only. Furthermore, this new method is cost effective and allows for quick prediction of blood parasitaemia that can be conducted in less than 15 min instead of requiring several hours to several days of technical time and labour. Furthermore, with no need to wait for flow cytometry confirmation, malaria disease progression can be assessed, moribund animals can be pinpointed, and animal deaths can be predicted with high confidence up to 8 days before they display clinical symptoms of the disease. Implementation of this new methodology almost totally eliminates finding the animals dead from severe malaria in the cages, as well as confirms the actual cause of animal death and maximizes the amount of data obtained from each study animal that would otherwise be lost.

## Methods

### Animals

Female ICR-CD1 mice weighing 20–25 g were purchased from Charles River Laboratories (Wilmington, MA). All animals were purchased from the same vendor to prevent possible genetic variations by using animals from a different colony that may interfere with the results. Facilities were kept under a 12 h light/dark period at a room temperature of 22 ± 2 °C and 40–70% relative humidity and air-conditioned with twenty air changes per hour. Filtered water and pelleted diet were provided ad libitum. Animal husbandry and room/cage cleaning and sanitation was provided in accordance with WRAIR/NMRC SOP No. 555 entitled ‘Husbandry of Rodents’.

### Modified Thompson test

Female ICR-CD1 mice weighing 20 to 25 g (Charles River, Inc.) were used throughout the modified Thompson test study. On the donor infection day, a vial of cryopreserved malaria blood (prepared as described below) was taken out of the liquid nitrogen tank, thawed by quickly submerging in warm water (37 °C), and immediately used for infection. Each of the two donor animals were infected by inoculating 200 µL of *P. berghei*-infected blood into the intra-peritoneal (IP) cavity by using a 27½ gauge needle (OpticsPlanet, Inc. Northbrook IL) and a 1 mL syringe. Four days later, blood parasitaemia of donor animals was measured using a Giemsa-stained thin blood smear as described below [23]. Approximately 0.5–0.7 mL of whole blood from donor animals was harvested via cardiac puncture using a 5/8 25 g needle (OpticsPlanet, Inc. Northbrook IL) and a 1 mL syringe, and the sample was then diluted in 1× PBS to the desired parasitaemia for infection [45]. Study mice were then injected IP with 200 µL of infected blood containing 1 × 10^5^ luciferase-expressing *P. berghei* infected erythrocytes. Experimental drugs were administered orally (PO) once daily for 3 days on days 3–5 post infection. Five mice were used per dose group and 6 different compound doses plus a vehicle control (0.5% hydroxyethyl cellulose (HEC)-0.1% Tween 80) and a positive control (mefloquine, WRAIR Repository, Rockville, MD) were tested in each study. Blood samples (3 µL) were taken initially on day 3 to determine the initial parasitaemia, and then once per week (on last weekday prior to the weekend) for 31 days. Parasitaemia was measured by using a flow cytometry system as described below. Temperatures, body weights, and behavioural observations were tracked daily on weekdays to determine when mice needed to be removed from study as a consequence of meeting euthanasia criteria specified in the main animal protocol and listed in Additional file 1 [21, 22]. Rectal temperatures were measured using an Oakton Acorn Temp JKT Thermocouple Thermometer (Cole-Parmer Lab Supplies, Vernon Hills, IL) with a resolution of 0.1 °F/C which was connected to a MLT1404 probe for mice (AD Instruments Inc., Colorado, Springs CO). Animal weights were measured using a Sartorius Cubis Semi-Micro Balance (Sartorius TCC, Arvada, CO) with 0.01 g sensitivity. Blood samples for parasitaemia determination were also taken prior to euthanizing the animals removed from study. Mice that were alive and tested negative for parasitaemia on day 31 post infection were considered cured.

### Preparation of cryopreserved malaria parasitized whole blood samples

Alsevier’s solution was used for cryopreservation of malaria parasites [23]. Briefly, 0.52 g analytic grade sodium chloride (Sigma-Aldrich Corp, St. Louis, MO) and 1 g tri-sodium citrate (Sigma-Aldrich Corp, St. Louis, MO) were dissolved in 100 mL of deionized distilled water (Sigma-Aldrich Corp, St. Louis, MO) by continuous stirring. To this mixture were later added 2.33 g of dextrose (Sigma-Aldrich Corp, St. Louis, MO) and 9.5 mL analytical grade glycerol (Sigma-Aldrich Corp, St. Louis, MO). The solution was stored at 4 °C until used. Parasitaemia of donor animals which were used for preparation of cryopreserved malaria whole blood samples was measured using Giemsa-stained thin blood smears as described below. Whole blood was harvested via cardiac puncture using a 25^5^/8 G needle (OpticsPlanet, Inc. Northbrook IL) and a 1 mL syringe [23]. Alsevier’s solution was then used to dilute the whole blood parasitaemia to 1%. Blood was distributed into 1 mL cryogenic storage vials (Sigma-Aldrich Corp, St. Louis, MO Cat # 366656) which were immediately placed in cryogenic storage boxes. Cryopreserved parasite stocks were stored in a liquid nitrogen tank to maximize parasite viability [23].

### Parasites

Luciferase and GFP-expressing *P. berghei* ANKA parasite strain (MRA-868) was obtained from MR4 [38]. Presently this strain resides with BEI Resources.

### Parasitaemia measurements using Giemsa-stained thin blood smears

Parasitaemia in donor mice was measured using Giemsa-stained blood smears as described in [23, 45], and [46]. In brief, the tails of the mice were gently wiped with 70% alcohol and allowed to dry. A 21G needle (OpticsPlanet, Inc. Northbrook IL) was then used obtain blood from the tails of donor animals. Five microliters (µL) of blood were taken from the puncture site using a 10 µL pipette. Once the blood volume was obtained, sterile gauze was applied with pressure to stop any blood flow from the mouse’s tail. A drop of blood was placed on a clean disposable microscope slide (Thermo Fisher Scientific, Waltham, MA), and another microscope slide was used as a spreader to prepare thin smears. The slides were allowed to air dry before the red blood cells (RBC) were fixed with methanol (Sigma-Aldrich Corp, St. Louis, MO) which was applied across the slide for approximately 5 s and then drained. Slides were allowed to air dry and then stained for 15 min using freshly prepared 10% Giemsa Modified Solution (Sigma-Aldrich Corp, St. Louis, MO) solution in distilled water. After staining, the slides were rinsed thoroughly in distilled water and allowed to dry before microscopy analysis. Olympus-BX43F Microscope with oil immersion (Cargille Labs, Cedar Grove, NJ) was used to count the parasites in the Giemsa-stained thin blood smears as described [23].

### Parasitaemia measurements using flow cytometry

Parasitaemia measurements using flow cytometry were measured as described in [29, 31]. In brief, 3 µL of blood was obtained as described above. The blood was transferred into 0.3 mL of 1% heparinized isotonic buffer (PBS saline). One millilitre of 0.04% of glutaraldehyde was added to fix each sample, and samples were incubated for 60 min at 4 °C followed by centrifugation at 450*g* for 5 min. The supernatant was removed by aspiration, and the cells were resuspended in 0.5 mL PBS buffer supplemented with 0.25% (v/v) Triton X-100 (Sigma-Aldrich St Louis, MO) for 10-min incubation at room temperature. After centrifugation, the permeabilized cells were suspended in 0.5 mL of RNase (Sigma-Aldrich St Louis, MO) at 1 mg/mL concentration and incubated for at least 2 h at 37 °C to ensure complete digestion of reticulocyte RNA. Twenty microliters of YOYO-1 dye (Invitrogen Corp. Carlsbad, CA) at a concentration of 2500 ng/mL was added to the 0.5 mL sample volume to create a final dye concentration of 100 ng/mL of YOYO-1. Parasitaemia was quantified using an FC500 MPL flow cytometer (Beckman Coulter, Fullerton, CA). The green photomultiplier tube and filter setting were used for these studies (520–555 nm filter settings for the green PMT and greater than 580 nm settings for the red PMT). Infected erythrocytes, uninfected erythrocytes, and leukocytes were gated on logarithmic forward/side dot plots.

### In vivo bioluminescence imaging of female ICR-CD1 mice infected with luciferase—expressing *P. berghei* parasites

Luciferin (d-Luciferin potassium salt, Xenogen, CA and Gold Biotechnology, St. Louis, MO), the luciferase substrate, at a concentration of 200 mg/kg was inoculated intraperitoneally (IP) into female ICR-CD1 mice using a 27½ gauge needle, 10 min before bioluminescence analysis. Animals were anaesthetized in a 2.5% isoflurane atmosphere (MWI Veterinary Supply, Harrisburg, PA) for 5 min and maintained in the imaging chamber for analysis. Emitted photons were collected by auto acquisition with a charge coupled device (CCD) camera [Perkin Elmer Spectrum In Vivo Imaging System (IVIS)] using the medium resolution (medium binning) mode. Analysis was performed after defining a region of interest (ROI) that delimited the surface of the affected area. Whole-body total photon emission was quantified with Living Image software (Xenogen Corporation, Alameda, CA), and results were expressed in numbers of photons/s.

### Correlation between whole-body bioluminescence signal, flow cytometry, and Giemsa-stained thin blood smears measured parasitaemia in donor female ICR-CD1 mice infected with luciferase-expressing *P. berghei* infected erythrocytes

In order to determine if there was a positive correlation between the three methods of determining parasitaemia in donor animals, a total of 16 mice were used to assess parasitaemia using whole-body bioluminescence and Giemsa-staining, and 16 female ICR-CD1 mice were used to assess parasitaemia using whole-body bioluminescence and flow cytometry. Whole-body bioluminescence signal measurement, Giemsa-stained thin blood smears and flow cytometry measurements were conducted as described above.

### Comparison between initial (day 3) parasitaemia in study animals in which whole-body parasite load or Giemsa-stained thin blood smears were respectively used to assess PLPP or measure parasitaemia in donor animals

Flow cytometry was used to measure initial parasitaemia on day 3 post infection in four modified Thompson test studies (a total of 160 ICR-CD1 mice) in which donor mice parasitaemia was assessed using Giemsa-stained thin blood smears. The same measurements were taken in four other modified Thompson test studies (a total of 160 ICR-CD1 mice) in which parasitaemia in donor animals was assessed by PLPP.

### Correlation between whole-body and brain parasite load in female ICR-CD1 mice infected with luciferase-expressing *P. berghei* infected erythrocytes

For this study, a total of 18 study mice that belonged to 8 different studies and that were due to be euthanized were randomly chosen. Whole-body luminescence signal was measured prior to euthanasia. Immediately after euthanasia, the brains of these mice were harvested, placed in the reading chamber of the IVIS instrument, and bioluminescence images were taken as described above.

### Evolution of flow cytometry measured parasitaemia and PLPP on day 1–7 post animal infection of ICR-CD1 mice infected with 1 × 10^5^ luciferase-expressing *P. berghei* infected erythrocytes

Thirty ICR-CD1 female mice were infected IP with 1 × 10^5^ luciferase-expressing *P. berghei* infected erythrocytes. Parasitaemia was measured for each mouse on days 1, 2, 3, 4, 6 and 7 post infections using flow cytometry. In addition, whole-body bioluminescence signal was measured and PLPP was calculated using Eqs. (2) and (3) for all mice on the same days. Three (3) and 8 (eight) ICR-CD1 mice had to be removed from the study respectively on days 6 and 7 post infection due to severe malaria symptoms. An unpaired t-test with Welch’s correction was used to compare differences between group means. A P-value < 0.05 was considered statistically significant (*P < 0.05).

### Statistical analysis

Linear and power regression model algebraic equations as well as R-squared coefficients were determined by using the Microsoft Excel statistical package. Statistical analysis of parasitaemia determined by flow cytometry and bioluminescence imaging in study animals from modified Thompson’s Test experiments was conducted using the Graph Pad Prism7 statistical software (unpaired t-test with Welch’s correction). *P* values ≤ 0.05 were considered statistically significant (*).

## Results

### Determination of luciferin kinetics and IVIS machine settings for measuring whole-body bioluminescence in ICR-CD1 mice infected with luciferase-expressing *P. berghei* infected erythrocytes

**Fig. 1:**
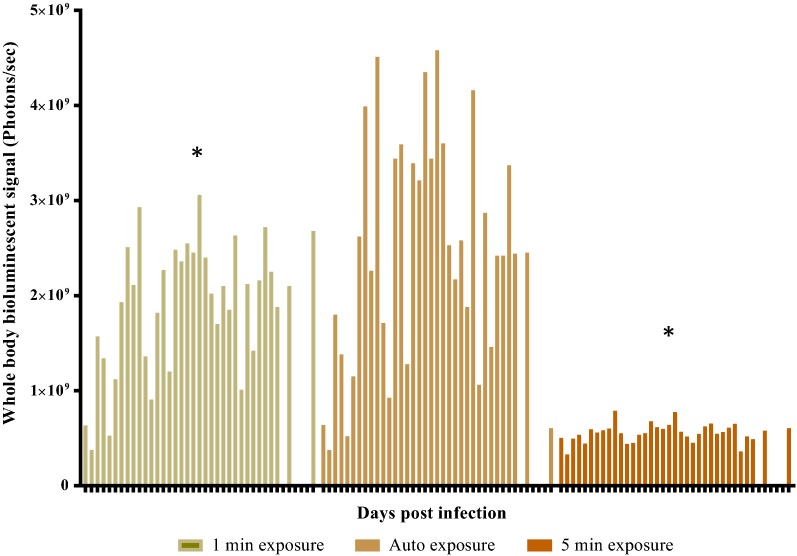
Determination of IVIS machine exposure time for determining full body bioluminescence measurements in ICR-CD1 mice infected with 1 × 10^5^ luciferase-expressing *Plasmodium berghei* infected erythrocytes. Ten ICR-CD1 female mice were infected IP with 1 × 10^5^ luciferase-expressing *P. berghei* infected erythrocytes. Data shown represents full body luminescence measured for all 10 mice on day 3–5 post infections and 7 mice on day 6 post infections. Three ICR-CD1 mice had to be removed from the study on day 6 post infections before the whole-body bioluminescence readings were taken because they became sick with malaria. The asterisk sign asterisk indicates presence of saturated images in one or more study mice. One and 5 min exposure, as well as auto exposure settings were used to measure whole-body bioluminescence signal

In order to assess the best time period for bioluminescence imaging after luciferin injection, 5 ICR-CD1 mice were infected IP with 1 × 10^5^ luciferase-expressing *P. berghei* infected erythrocytes. Five days post-infection, mice were injected with 200 mg/kg luciferin administered IP, anesthetized, placed in the imaging chamber of the Perkin Elmer IVIS instrument, and consecutive bioluminescence readings using the auto option were taken every minute starting at minute 6 post luciferin injection. Initially, the bioluminescence signal increased before reaching a plateau approximately 10–12 min post-luciferin injection and started diminishing soon thereafter (Additional file 2). Therefore, the bioluminescence imaging for all subsequent experiments were conducted 10 min after luciferin injection to maximize bioluminescence signal and minimize exposure times. To determine the exposure time that should be used for measuring full body bioluminescence in study mice, 10 ICR-CD1 mice were injected IP with 1 × 10^*5*^ luciferase-expressing *P. berghei* infected erythrocytes. Full body bioluminescence was measured in the same 10 mice on days 3, 4, 5, post infection, and 7 mice were assessed on day 6 post infection (3 of 10 ICR-CD1 mice were euthanized in the morning of day 6 post infection due to severe malaria). In order to be able to detect and accurately quantify both low and high parasitaemia in study animals, exposure times of 1 min and 5 min were used in addition to the recommended and widely used auto exposure option. Experimental results are shown in Fig. 1. Auto exposure is by far the most sensitive option for the detection and accurate measurement of full body bioluminescence signals. Not only did the use of 1 min and 5 min exposure times fail to increase overall sensitivity, but most of the full body luminescence values obtained when using long exposure times were not accurate due to saturation of the bioluminescence images. Total body bioluminescence signal measured through the “auto” option on days 3–6 post infection were 4–5 times higher than the signals measured by using a 5 min exposure time and 2–3 times higher than the signal measured using a 1 min exposure time (Fig. 1). In addition, the optimal binning settings for the in vivo imaging system (IVIS) for all drug testing studies was examined through iterative testing. The most effective setting was found to be medium binning. As a result, the optimal IVIS machine settings for all whole-body bioluminescence signal measurements were found to be medium binning and auto exposure.

### Disease progression and the associated whole-body bioluminescence signal in mice during a modified Thompson test study

**Fig. 2:**
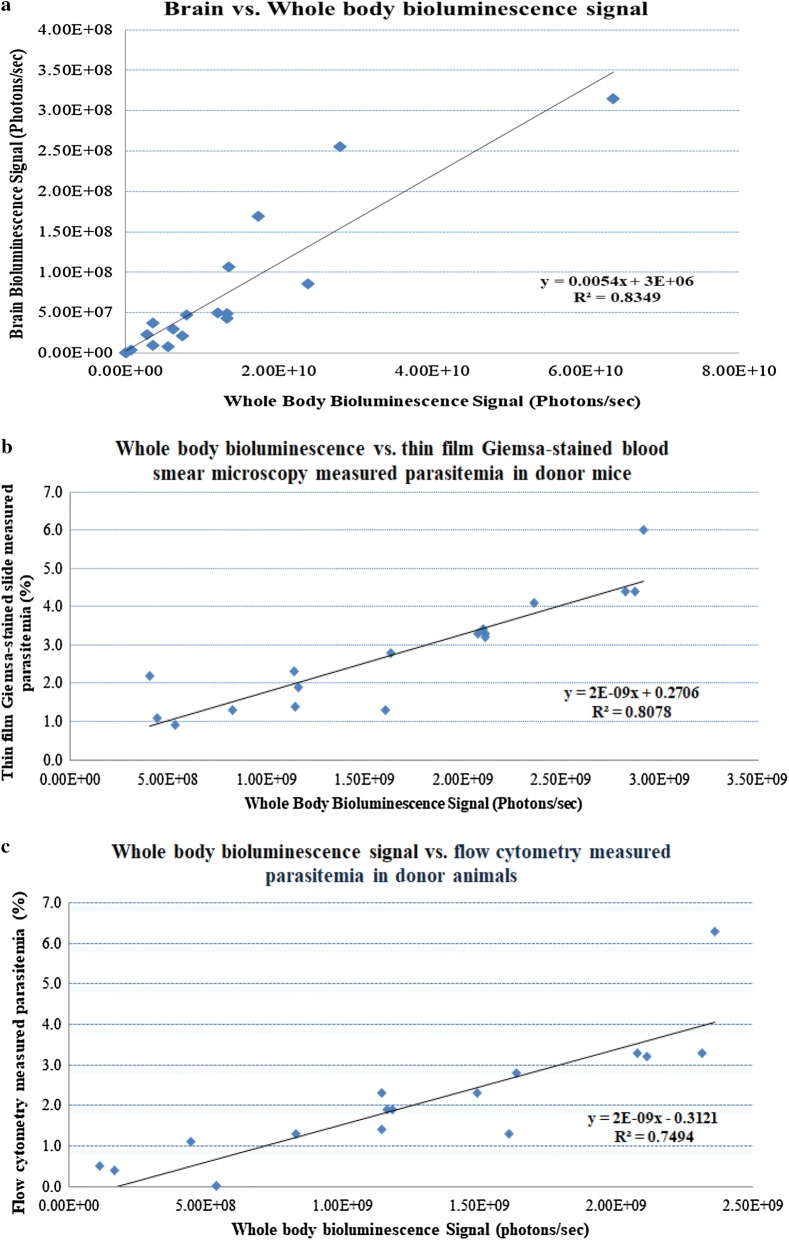
Correlation between whole-body and brain parasitaemia (**a**), whole-body bioluminescence signal and Giemsa-stained thin blood smears (**b**), flow cytometry and whole-body bioluminescence signal (**c**) in donor female ICR-CD1 mice infected with luciferase-expressing *P. berghei* infected erythrocytes. A total of 18, 16, and 16, female ICR-CD1 mice were used respectively in the studies **a**–**c**. Linear regression coefficients between variables were determined using the Microsoft Excel statistical package

After ICR-CD1 mice were infected with 1 × 10^5^ luciferase-expressing *P. berghei* infected erythrocytes, a period of parasite growth is observed which is reflected in the blood parasitaemia. Starting from day 1 after animal infection, whole-body bioluminescence increased in study mice up to day 7 post-infection (Additional file 3). Untreated mice started showing signs of malaria on days 4–6, and some of them were euthanized due to severe malaria by day 7 post-infection. This timeline is comparable with the disease progression and animal death described for *P. berghei* infection of mice in the literature [23]. The intensity of the in vivo bioluminescence signal emitted by infected ICR-CD1 mice reflects the high number of parasites in their blood and brain (Additional file 4). Whole-body and brain bioluminescence signal were measured in 18 mice that were randomly chosen and belonged to 8 different studies. As shown in Fig. 2a, a very strong linear correlation was observed between the bioluminescence signal and the number of parasites in the blood and brain (R^2^ = 83.4%).

### Correlation between parasitaemia determined using whole-body bioluminescence signal, flow cytometry, and Giemsa-stained thin blood smears in female ICR-CD1 donor mice infected with 1 × 10^5^ luciferase-expressing *P. berghei* infected erythrocytes

In our model, blood parasitaemia increased in a predictable way in female ICR-CD1 mice infected with 1 × 10^5^ luciferase-expressing *P. berghei* infected erythrocytes. Four days after being infected via IP injection with 200 µL of cryopreserved blood infected at a 1% parasitaemia, Giemsa-stained thin blood smears and whole-body bioluminescence signals were used to determine parasitaemia in 16 donor mice used to conduct 7 different modified Thomson test studies. A strong linear correlation was observed between microscopy measured parasitaemia and whole-body bioluminescence signal (R^2^ = 80.7%) in donor mice on day 4 post infection with cryopreserved blood (Fig. 2b). The line of regression that predicts the parasitaemia value based on total body bioluminescence signal is:

1 $$ {\text{y }} = \, 2{\text{E}}^{ - 9} {\text{x }} + 0.2706 $$

where x = measured whole-body bioluminescence signal and y = PLPP in donor mice.

In similar studies, 3 μL of blood was collected from the tail tip of 16 female ICR-CD1 donor mice used to conduct 5 different modified Thomson test studies. Parasitaemia was measured using flow cytometry. Whole-body bioluminescence signals were also assessed in all 16 mice. There was a strong linear correlation between parasitaemia measured by flow cytometry and parasitaemia assessed using full body bioluminescence (R^2^ = 74.9%) (Fig. 2c). In a proof of concept study, flow cytometry was compared with Giemsa-stained thin blood smears measured parasitaemia in 17 female ICR-CD1 mice. Even though there was a strong linear correlation between these two methods of determining parasitaemia in donor animals (R^2^ value = 79.3%), use of the Giemsa-stained thin blood smears yielded higher parasitaemia readouts compared to flow cytometry in donor animals.

### Comparison between initial (day 3) parasitaemia (P0) in study animals in which whole-body bioluminescence signal or Giemsa-stained thin blood smears were used to determine parasitaemia in donor animals

**Fig. 3:**
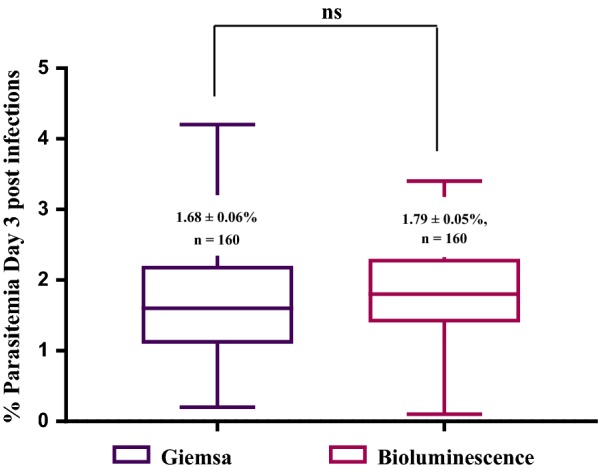
Comparison of the initial parasitaemia (P0) in study mice in which the parasitaemia of donor animals was measured using Giemsa-stained thin blood smears *versus* whole-body luminescence. The graph represents P0 parasitaemia in 160 study mice in which the parasitaemia of donor animals was measured using Giemsa-stained thin blood smears vs. 160 mice in which whole-body parasitaemia was used to predict parasitaemia in donor mice using Eq. (1). Error bars represent the ± SEM parasitaemia for each experimental condition. An unpaired t-test with Welch’s correction was used to determine significant differences between study groups. Values P < 0.05 were considered statistically significant

P0 parasitaemia was measured in 160 study mice used to conduct 4 different modified Thompson test studies in which donor mice parasitaemia was determined using Giemsa-stained thin blood smears as well as in 160 mice in which donor mice parasitaemia was predicted using the linear correlation Eq. (1). The P0 parasitaemia values obtained on day 3 post-infection were not statistically different from each other (Fig. 3).

### Use of whole-body bioluminescence signal measurement to assess blood parasitaemia in female ICR-CD1 study mice infected with 1 × 10^5^ luciferase-expressing *P. berghei* infected erythrocytes

**Fig. 4:**
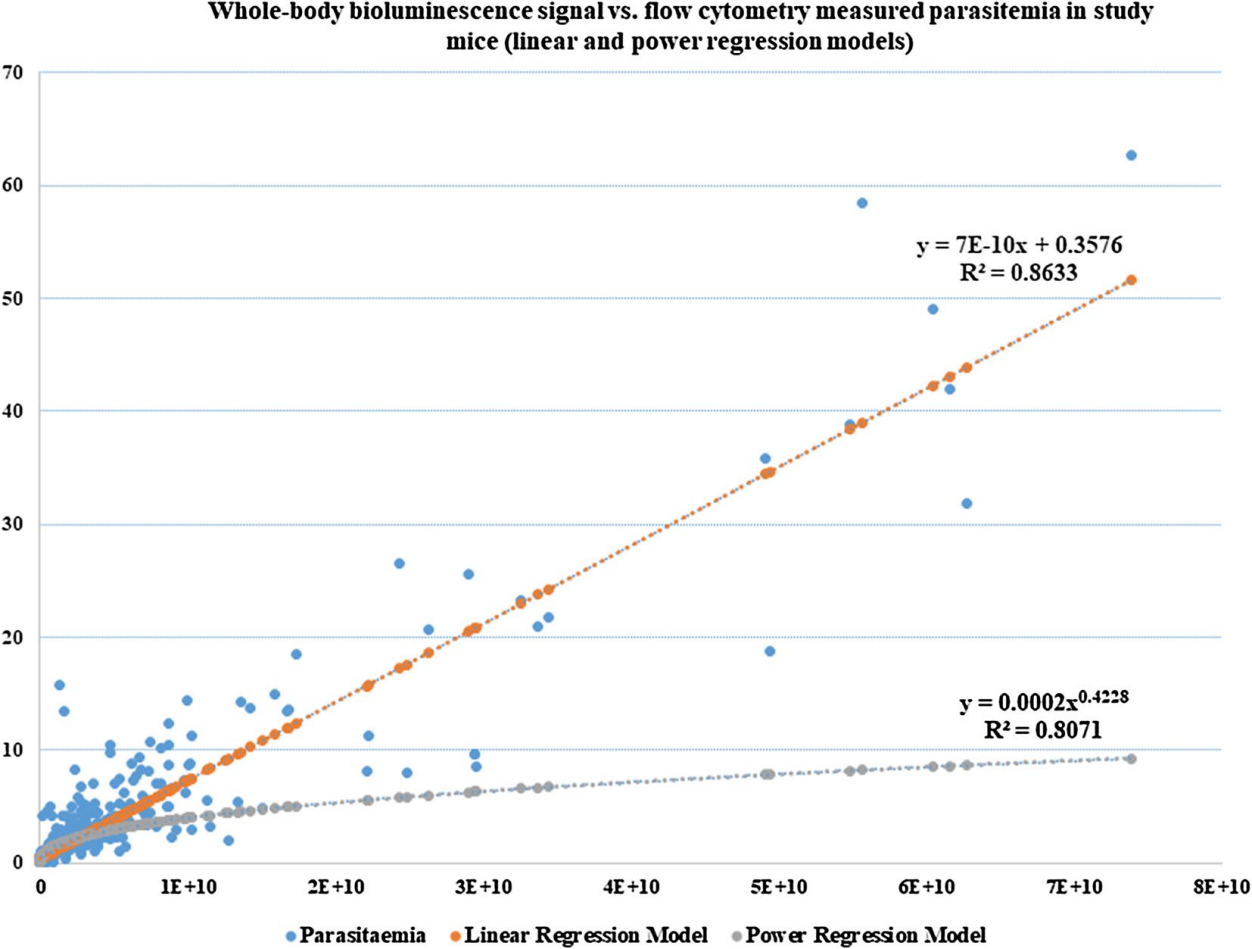
Correlation between the whole-body bioluminescence signal and flow cytometry measured parasitaemia in female ICR-CD1 study mice. Whole-body bioluminescence signal and flow cytometry measurements were obtained from 240 study mice associated with six different modified Thompson test studies. A total of 702 data points were obtained throughout the duration of these studies. Linear and regression equations and correlation coefficients were determined using the Microsoft Excel statistical package

In vivo whole-body bioluminescence signal and flow cytometry measured parasitaemia were simultaneously assessed in 240 female ICR-CD1 mice associated with six different modified Thompson test studies. These mice belonged to the vehicle control (VC) groups, the positive control groups (which were given 3 doses of 90 mg/kg mefloquine) as well as several potential antimalarial drugs which were administered at doses ranging from 20 to 160 mg/kg. Vehicle control as well as the positive and experimental drugs were formulated and administered as described in the Thompson test experimental design. A total of 702 data points were collected throughout the duration of the studies. Flow cytometry measured parasitaemia ranged from 0.1 to 0.2 (which is considered background parasitaemia) to 62.7% which was the highest value overall obtained in these six studies. The whole-body bioluminescence signal ranged from 1.15 × 10^5^ to 7.39 × 10^10^ photons/s (Fig. 4). Both linear and power regression models were used to fit the 702 data points obtained from study mice.

The linear regression equation obtained using the original data was:

2 $$ {\text{y }} = {\text{ 7E}} - 10{\text{x }} + \, 0. 3 5 7 6 $$

where x = measured whole-body bioluminescence signal and y = PLPP in study mice.

The R-squared values for the linear regression was 86.3%, which showed that the linear regression is an accurate model for predicting parasitaemia. Since most of the animals with high parasitaemia are euthanized early in the study due to clinical observations, the majority of the study animals had low parasitaemia. As a result, the data for both bioluminescence signal and the respective PLPP’s have extreme values, which generate high influence points. The normal distribution and equal variance assumption of linear regression are not valid (Fig. 4). Hence the logarithm transformation was used for both PLPP and bioluminescence signal. While the linear regression gives the same weight to all data no matter how big or small bioluminescence signals, by using logarithm, the non-linear model gives more weight on the small values to avoid the large influence on the model from a few extreme large bioluminescence signals. The estimated equation of the regression by using logarithm for this set of data is:

3 $$ {\text{y }} = \, 0.000 2 {\text{x}}^{0. 4 2 2 8} $$

Equation (3) is a power regression model in which x = measured whole-body bioluminescence signal and y = PLPP in study mice.

The R-squared values for the power regression was 80.7%, which is similar but just a little lower than 86.3%, the R-square obtained by using linear regression. As mentioned above, in the power regression model, more weight is given to the to the small bioluminescence signal values and larger residuals are observed for the high bioluminescence signal values (Fig. 4). As a result, Eq. (3) makes better predictions of PLPP for lower bioluminescence signal, and the linear model gives better predictions of PLPP for larger bioluminescence signal (Fig. 4).

### Evolution of parasite load predicted parasitaemia (PLPP) and flow cytometry measured parasitaemia in ICR-CD1 mice infected with 1 × 10^5^ luciferase-expressing *P. berghei* ANKA infected erythrocytes

**Fig. 5:**
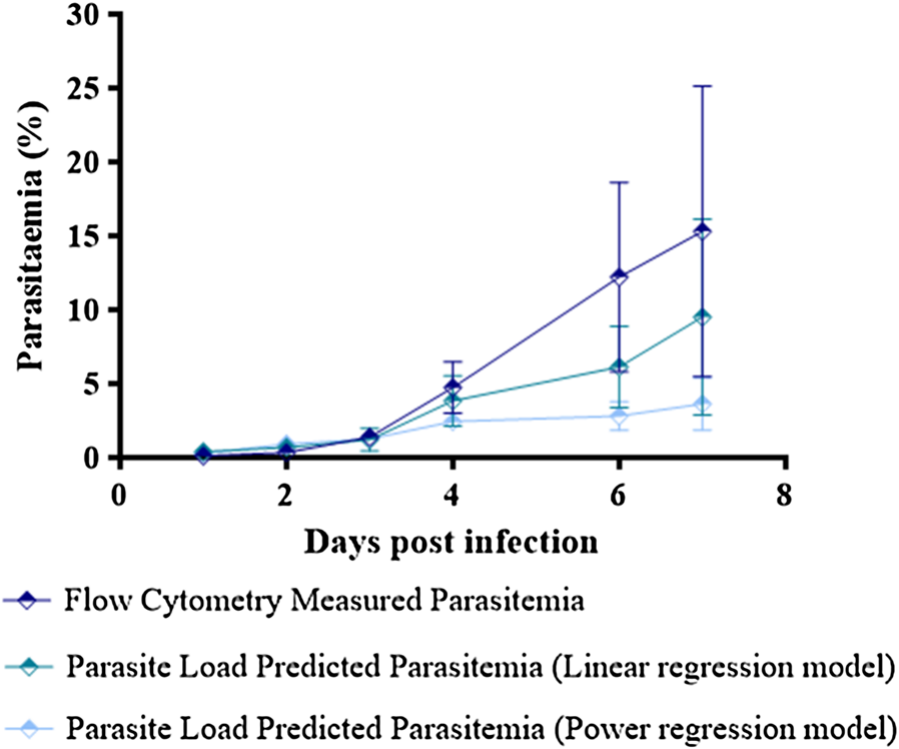
Evolution of parasite load predicted parasitaemia (PLPP) and flow cytometry measured parasitaemia in ICR-CD1 mice infected with 1 × 10^5^ luciferase-expressing *P. berghei* ANKA infected erythrocytes. Thirty ICR-CD1 female mice were infected IP with 1 × 10^5^ luciferase-expressing *P. berghei* infected erythrocytes. Data shown represents evolution of PLPP calculated using the linear and power Eqs. (2) and (3) as well as flow cytometry measured parasitaemia in all 30 mice on days 1, 2, 3, 4, 6, and 7 post infections. Each data point represents mean ± standard error for the PLPP and flow cytometry measured parasitaemia for 30, 30, 30, 30, 27 and 19 ICR-CD1 mice respectively on days 1, 2, 3, 4, 6, and 7 post infections. Three (3) and 8 (eight) ICR-CD1 mice had to be removed from the study respectively on days 6 and 7 post infections due to severe malaria symptoms. An unpaired t-test with Welch’s correction was used to compare differences between group means. A P-value < 0.05 was considered statistically significant (*P < 0.05)

In order to confirm that PLPP [predicted using Eqs. (2) and (3)] is comparable with the parasitaemia readouts using flow cytometry, we followed both those variables in 30 ICR-CD1 mice infected with 1 × 10^5^ luciferase-expressing *P. berghei* infected erythrocytes on days 1–7 post animal infection. Results are shown in Fig. 5.

When using the linear Eq. (2), on day 1 post infection, the mean PLPP was 0.38 ± 0.002 (0.02% above the limit of detection for this model) compared to 0.11 ± 0.006% to the flow cytometry measured parasitaemia, which, as mentioned above, is within the 0.1–0.2% background for this methodology. On day 2 post infections PLPP and flow cytometry measured parasitaemia were respectively 0.69 ± 0.03% and 0.32 ± 0.01% which shows that PLPP is a more sensitive method for predicting low blood stage parasitaemia compared to flow cytometry. On day 3 post infections PLPP and flow cytometry measured parasitaemia were respectively 1.22 ± 0.14 and 1.39 ± 0.10% and on day 4 these values were respectively 4.73 ± 0.31 and 3.82 ± 0.31%. As seen in Fig. 5, after day 3 post infections, flow cytometry measured parasitaemia in study mice increased at a much higher rate compared to PLPP, which shows that PLPP assessed using the linear Eq. (2) tends to underestimate flow cytometry parasitaemia readouts above 4–5%. On day 6 post infections PLPP and flow cytometry measured parasitaemia were respectively 5.93 ± 0.54 and 12.21 ± 1.23%. On this day 3 ICR-CD1 mice had to be removed from the study due to severe symptoms of malaria disease. On day 7 post infections PLPP and flow cytometry measured parasitaemia were respectively, 9.52 ± 1.51% and 15.32 ± 2.25%. Eight (8) ICR-CD1 mice were removed from the study on day 7 post infections because of severe malaria symptoms. An unpaired t-test with Welch’s correction which was used to determine statistical difference between groups showed that PLPP values were not statistically significant from flow cytometry measured parasitaemia on days 2, 3, 4, and 7 post infections. The lack of statistical difference between PLPP and flow cytometry measured parasitaemia on day 7 post infections is most probably due to high variability within both these groups (Fig. 5).

When using the power regression Eq. (3), the mean PLPP’s on days 1, 2, 3, 4, 6, and 7 post infection were respectively 0.30 ± 0.02, 0.91 ± 0.03, 1.26 ± 0.1, 2.42 ± 0.10, 3.03 ± 0.18 and 4.02 ± 0.31%. Flow cytometry measured and PLPP obtained using the exponential regression parasitaemias were not statistically significant with each other on day 2 post animal infection. Power regression was shown to be more sensitive than the linear regression model for PLPPs that correspond to flow cytometry measured values up to 1.39%.

### Limit of detection of bioluminescence signal in female ICR-CD1 mice infected with luciferase-expressing *P. berghei* infected erythrocytes

Bioluminescence signal emitted by the *P. berghei* parasites represents light intensities over the ICR-CD1 mice body surface area, with red indicating the most intense signal and blue indicating the weakest signal (Additional file 4). The smallest bioluminescence signal recorded for non-infected animals (no signal over the body surface area) is 1.10 × 10^5^ photons/s, but similar values are also present in study mice treated with the positive control and/or other strong anti-malarial drugs. At low parasitaemia, body thickness can quench the bioluminescence signals emitted from parasites present in the blood; however, the parasites were usually visible in the blood at the thin tails of infected mice (Additional file 5). As stated above and seen in Additional file 5, the lowest level of bioluminescence in which the parasites were visible in the IVIS system is 1.96 × 10^6^ photons/s. Based on the linear Eq. (1), the predicted lower limit of detection for the donor mice is 0.27%. Similarly, the predicted lower limit of detection for study mice based on the linear (2) and power (3) regression models is respectively 0.36% and 0.09%. A lack of a bioluminescence signal suggested that the parasite load was non-existent or below the limit of detection. The PLPP of mice in which bioluminescence signal is < 1.96 × 10^6^ is marked as zero (0%).

### Use of whole-body bioluminescence signal measurement to follow disease progression and predict animal death in the modified Thompson test studies

Based on historical data, after day 6 post infection, if the observed parasitaemia by flow cytometry was ≥ 1.50% all *P. berghei* infected ICR-CD1 mice without exception demonstrated a continuous increase of parasitaemia until the animal was ultimately euthanized due to clinical observations or found dead in the cage because of severe malaria. Recent observations showed that if after day 6 post infection the overall whole-body bioluminescence signal reached or exceeded 1.5 × 10^9^ photons/s, study mice without exception had to be euthanized sooner or later because of signs of severe malaria. As mentioned above, both linear and power regression models can be used with high confidence to predict parasitaemia in study mice (R^2^ values were respectively 86.3 and 80.7%). Still, the power regression model provided a much lower limit of detection compared to the linear regression model (0.09 vs. 0.36%). In addition, parasitaemia readouts by flow cytometry up to 1.39% were not statistically different from PLPP predicted by utilizing the power regression model (1.39 ± 0.10% vs. 1.26 ± 0.10, P < 0.0001). Based on Eq. (3) the 1.5 × 10^9^ photons/s value corresponds to a PLPP of 1.52%. Therefore, the power regression model (Eq. 3) was chosen to assess low PLPPs and to establish the euthanization criteria in study mice. This new method provided a means to rapidly predict parasitaemia in study animals which makes it possible to remove animals from study earlier than previously. To account for testing long half-life anti-malarial drugs and given the fact that all mice with a 1.5% parasitaemia did not show any behavioural signs of malaria disease, nor had decreased core body temperature or weight loss, ICR-CD1 mice that reached threshold PLPP of 1.52% on day 5 (last day of treatment) were not euthanized. A confirmatory whole-body bioluminescence reading was taken on day 6 and 7 post infection, and the animal was euthanized after confirmation that parasitaemia had continued to increase and/or other signs of disease were present. Starting from the second week of the Thompson test studies, if the PLPP was ≥ 1.52%, a confirmatory whole-body bioluminescence signal was taken 4–5 h later and the animal was euthanized. Observations showed that by using these criteria animals could be removed from study 3–8 days before they would have otherwise done based on behavioural signs of disease and reduction in body weight and core temperature. An example is shown in Additional file 6. Mice 1–5 belonged to a study group which was given 20 mg/kg of a potential antimalarial compound on days 3, 4, and 5 post infections with 1 × 10^5^ *P. berghei* infected RBC’s. Based in the new criteria for using PLPP to euthanize study mice as soon as the bioluminescence signal reached ≥ 1.5 × 10^9^ photons/s, mouse 1, 3, 4, and 5 could have been euthanized on day 10 post infection. Instead, based on criteria mentioned in Additional file 1, mouse 1, 4, and 5 were euthanized on days 18, 13, and 13 post infections. Based on the same criteria, mouse #2 could have been euthanized on day 12; instead this mouse was FDIC on day 18 post infections.

Recent observations have shown that there is a 2–4 days delay in order for the bioluminescence signal to increase from background (10^5^–10^6^) to the 10^9^ photons/s level. Therefore, in order to be able to diagnose animals that developed clinical malaria over the weekend, the whole-body bioluminescence signal was measured in all study mice every Monday morning and anytime during the week when other signs of malaria disease were present. To determine disease progression and prevent animals from being found dead in the cage over the weekend, a second measurement of whole-body bioluminescence signal for each study animal was also taken every Friday morning. If a bioluminescence signal well above the lower limit of quantitation of 1.96 × 10^6^ was detected, the whole-body bioluminescence signal was measured again on Friday afternoon and a decision was made about whether the animal needed to be euthanized or not.

When using the linear regression model, the cut off value of 1.5 × 10^9^ photons/s corresponds to a 1.41% PLPP, which is close to the 1.52% PLPP assessed using the power regression model. As mentioned above, even though both regression models underestimate parasitaemia > 1.39% measured by flow cytometry, the linear regression model is more accurate for assessing higher PLPP’s; when using this model PLPPs were not statistically different from flow cytometry parasitaemia readouts up to 4.73%. As a result, if the whole-body bioluminescence signal is > 1.5 × 10^9^ photons/s, PLPP can be assessed using the linear regression Eq. (2). Overall, these models can predict with very high confidence parasitaemia values 0.09–4.73%.

## Discussion

Accurate measurement of blood parasitaemia in donor and study animals in rodent malaria models is of great importance since it is directly related to the assessment of the schizonticidal blood efficacy of potential anti-malarial compounds. The new methodology presented here uses whole-body luminescence signal measurements and algebraic formulas for predicting parasitaemia in donor and study mice and refines the modified Thompson test model of rodent malaria. Prior to this innovation, Giemsa-stained thin blood smears measurements was used to assess parasitaemia in donor mice. As shown in this study, in female ICR-CD1 mice there is a strong linear correlation between parasitaemia predicted by whole-body bioluminescence signal and parasitaemia measured using Giemsa-stained thin blood smears. In addition, initial blood parasitaemia measurements on the first day of study resulting from microscopy and parasitaemia values predicted through PLPP in donor mice were not statistically different. The new approach of using PLPP in donor animals required only 15 min of time compared to the Giemsa microscopy procedure which required several hours of technician time and highly trained technical staff. This new method of predicting parasitaemia has also successfully replaced flow cytometry procedures that require several days of intermittent work to yield data. In addition, this new method is by far the fastest one for predicting parasitaemia in rodent malaria studies, does not require highly trained technical staff, provides an accurate prediction of parasitaemia, and reduces per diem costs.

The ability to predict animal death with accuracy as soon as study goals have been fully achieved and the use of preemptive euthanasia to reduce unnecessary terminal pain and distress in experimental animals is of great importance and one of the most significant pillars of the 3 R’s of animal use in research. The new method of using PLPP can be used to predict animal death from malaria and to remove sick animals from study 3–8 days before they would have been otherwise removed based on parameters such as decreased body core temperature and loss of body weight. In addition, based on a review of historical data, a progression to death has been shown for animals whose blood parasitaemia exceeds 1.50%. The same is true for PLPPs; if after day 6 post infection the overall whole-body bioluminescence signal reached or exceeded 1.5 × 10^9^ photons/s, study mice without exception had to be euthanized sooner or later because of clinical manifestations of malaria disease. Based on Eq. (3), that value corresponds to a PLPP of 1.52%. In most cases, female ICR-CD1 mice with a 1.52% blood parasitaemia do not display any of the typical signs of malaria disease. One of the biggest benefits of this new technique is the ability to predict animal death with confidence, in many cases more than a week before the animal has to be euthanized or, in the worst scenario, are found dead in cage. If needed, the linear and power regression models can be combined to accurately assess PLPPs that are not statistically significant to flow cytometry readouts up to 4.73%.

The new methodology for rapid visualization and quantitation of *P*. *berghei* parasites has eliminated the loss of study data due to blood sample lysis, minimized false positive removals of animals from study, and has radically reduced the number of animals found dead in cage. In some cases, study mice die unexpectedly and are found dead in cage without displaying any behavioural signs of malaria disease, no recent measurable weight loss, and/or low core body temperatures. In these cases, the opportunity to obtain a final blood sample is missed, and a confirmation that the animal died of malaria cannot be made. Given the historical knowledge of disease and parasitaemia progression in this model over a 2–4 day period, parasitaemia predictions can be scheduled in a way to insure identification of parasitaemia increase that would previously not have been noted. Moreover, the ability to rapidly quantitate parasitaemia affords the means to minimize removal of animals from study due to false positive criteria which jeopardizes statistical power, confounds study data, and might result in a study repeat requirement.

Overall, these models can predict with very high confidence parasitaemia values 0.09–4.73%. Only pitfall of this method is the fact that despite the optimal correlation coefficients for both regression models, the linear and power regression methods underestimate parasitaemia respectively over 4.73% and 1.39% as measured by flow cytometry. Given that flow cytometry can accurately measure parasitaemia as high as 60.00% and sometimes higher, this is a drawback to this method. A plausible explanation to the tendency for underestimating parasitaemia is that the IVIS instrument may not be accurate in quantitating higher light intensities, even when the auto option is used. Nonetheless, this limitation is not relevant when considering its use in an infection model, such as the Thompson test where the increase of parasitaemia above the 1.5% threshold level uniformly leads to death.

The cost benefits from paying less for routine animal care expenses more than offsets the cost of luciferin, the only reagent required for whole-body luminescence assessment. The cost of luciferin used for assessing whole-body bioluminescence signal in a mouse is approximately US $1. Taking in consideration that sick mice are now removed from the study 3–8 days earlier than before, the cost to use this new method is more than offset by the per diem cost of $0.77 per mouse per day. Moreover, use of whole-body bioluminescence signal for quantifying parasitaemia is a one-step procedure that significantly reduces labour costs as well as eliminates the need for several expensive reagents that are used in flow cytometry.

Future directions for this research include utilizing these findings in other rodent malaria models such as the in vivo drug resistance studies and assessment of the mode of action of anti-malarial drugs [9, 47]. This innovation will provide an array of benefits for animal testing, and programmes engaging in high volume malaria drug testing in vivo will benefit by adopting this method.

## Conclusions

The new method described in this manuscript is a one-step procedure that uses the whole-body bioluminescence signal emitted by donor and study mice to assess the parasite load predicted parasitaemia in a rodent model of malaria drug discovery, the modified Thompson test. It provides for a fast, simple, and accurate prediction of parasitaemia as well as real-time following of the disease progression, eliminates false positive results and provides the means to refine the modified Thompson test through early removal of sick animals as soon as the study objective has been achieved, in many cases well before the clinical signs of disease are present. These findings can be applied to improve other rodent malaria models of in vivo drug discovery.

## Additional files

**Additional file 1.** Factors for determining animal removal from the study. If a mouse gets a score of 4 or greater, the animal is removed from the experiment and euthanized after consulting the staff veterinarian.

**Additional file 2.** Luciferin kinetics in ICR-CD1 mice. Luciferin was administered at 200 mg/kg IP to four female ICR-CD1 mice on day 5 post infection with 1 × 10^5^ luciferase-expressing *P. berghei* infected erythrocytes. Bioluminescence measurements were taken every 1 min starting at 6 min after luciferin administration. Data points represent the mean bioluminescence signal ± SEM for a total of four BALB/c mice for each data point.

**Additional file 3.** Evolution of whole-body bioluminescence signal in female ICR-CD1 mice infected with 1 × 10^5^ luciferase-expressing *Plasmodium berghei* infected erythrocytes. Thirty animals were infected with 1 × 10^5^ luciferase-expressing *P. berghei* infected erythrocytes on day 0. Whole-body bioluminescence signal was measured on day 1–7 post animal infections respectively on 30, 30, 30, 30, 30, 28, and 24 ICR-CD1 mice, as some animals started being euthanized because of severe malaria on day 6 post infections. The luciferase substrate luciferin was inoculated intraperitoneally (IP) into female ICR-CD1 mice at a concentration of 200 mg/kg, 10 min before bioluminescence analysis. Animals were anaesthetized in a 2.5% isoflurane for 5 min and maintained in the imaging chamber for analysis. Emitted photons were collected by auto acquisition using an IVIS Spectrum instrument. Analysis was performed after defining the animal’s whole-body as a region of interest (ROI). Whole-body total photon emission was quantified using the Living Image software and results were expressed in numbers of photons/s.

**Additional file 4.** Luciferase-expressing *Plasmodium berghei* infected erythrocytes are present in the blood and brain of female ICR-CD1 mice infected with *P. berghei* parasites. Female ICR-CD1 mice infected with 1 × 10^5^ luciferase-expressing *P. berghei* infected erythrocytes were injected IP with 200 mg/kg luciferin 10 min before analysis. Whole-body luminescence measurements were taken 10 min post luciferin injections in 10 ICR-CD1 mice. Mice were euthanized immediately thereafter, brains were harvested, and bioluminescence signal was measured. Additional figure 4A shows the bioluminescence signal in the bodies and brains of 5 mice respectively before and after they were euthanized because of severe malaria. Additional figure 4B shows bioluminescence signal in the bodies and brains of 5 mice that belonged to the positive control (mefloquine) treated group respectively before and after they were euthanized on day 31 post infections which is the last day in the study. The photon intensity numbers in the brighter areas of the image (red or bright yellow) are greater than those in the areas with a dimmer green or blue color, where fewer photons were detected.

**Additional file 5.** Limit of detection of the bioluminescence signal in ICR-CD1 study mice infected with luciferase-expressing *Plasmodium berghei* parasites. Female ICR-CD1 mice infected with 1 × 10^5^ luciferase-expressing *P. berghei* infected erythrocytes were injected IP with 200 mg/kg luciferin. Whole-body bioluminescence as well as flow cytometry parasitaemia measurements were taken simultaneously. The bioluminescence signal represents the light intensities over the body surface area. Red and bright yellow represent the most intense signal, followed by green and then blue, which represents the weakest signal. The 20 mice showed in this figure had no detectable parasitaemia measured through flow cytometry. Luciferase-expressing *P. berghei* parasites were visible in the tails of eight ICR-CD1 mice.

**Additional file 6.** Use of whole-body bioluminescence signal measurement to animal death in the modified Thompson test studies. Mice 1–5 belonged to a study group which was given 20 mg/kg of a potential antimalarial compound on days 3, 4, and 5 post infections with 1 × 10^5^ *P. berghei* infected RBC’s. Whole-body bioluminescence values marked in green show the day in which these mice could have been euthanized based in the new criteria for using PLPP to predict animal death in study mice (bioluminescence signal ≥ 1.5 × 10^9^ photons/s). Cells marked in yellow show the day in which these mice were euthanized based on euthanization criteria described in Additional file 1.

## Abbreviations

PLPP: parasite load predicted parasitaemia
RBC: red blood cells
IVIS: in vivo imaging system
FDIC: found dead in cage
