# Supplementary figures and images for: Updating the modified Thompson test by using whole-body bioluminescence imaging to replace traditional efficacy testing in experimental models of murine malaria
Source: Malar J. 2019 Feb 15;18:38. doi: 10.1186/s12936-019-2661-x (PMC6376706; doi:10.1186/s12936-019-2661-x)

## Slide 1
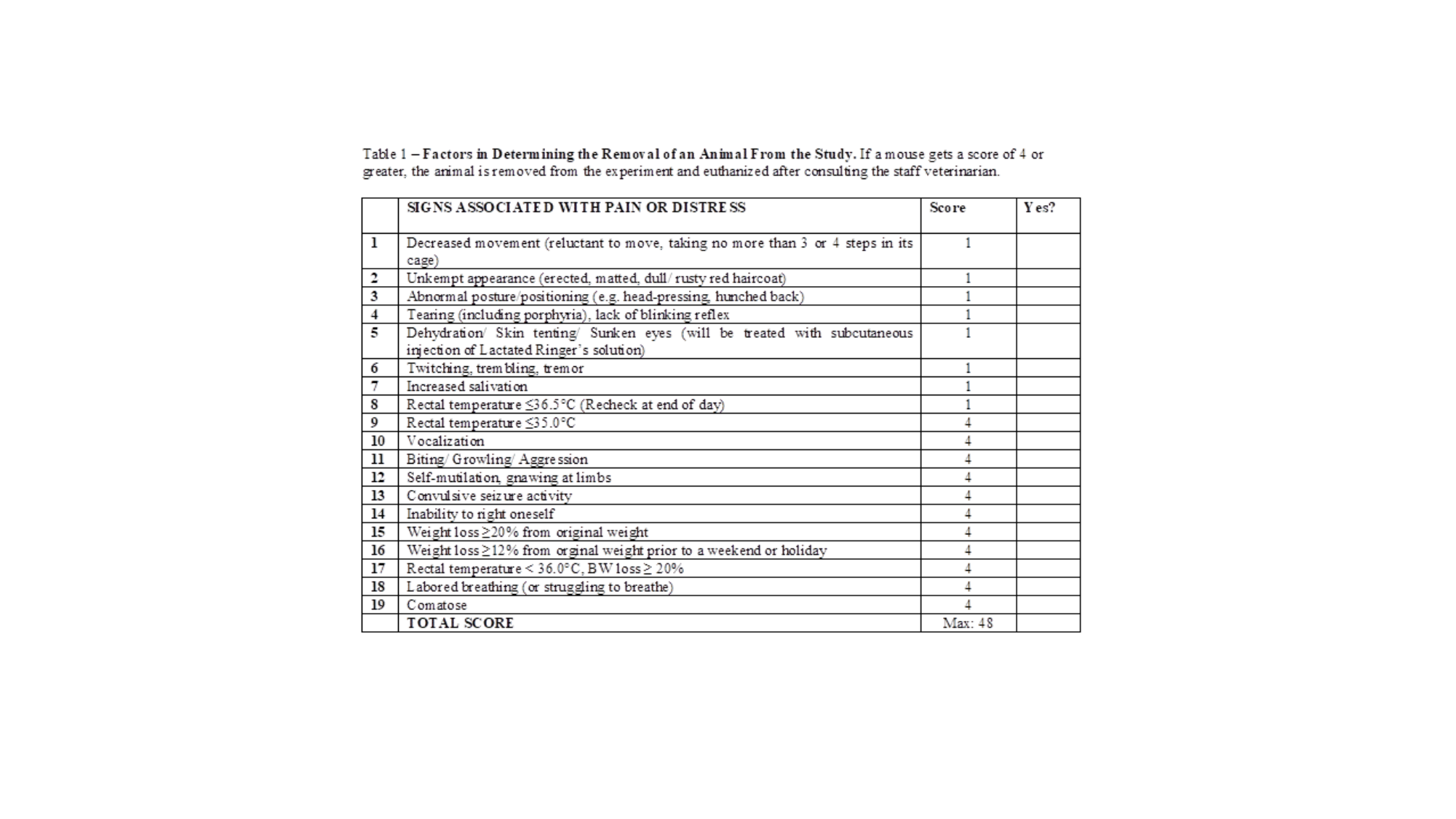

Supplement: Supplementary file 1 — Additional file 1. Factors for determining animal removal from the study. If a mouse gets a score of 4 or greater, the animal is removed from the experiment and euthanized after consulting the staff veterinarian. [file 12936_2019_2661_MOESM1_ESM.pptx]

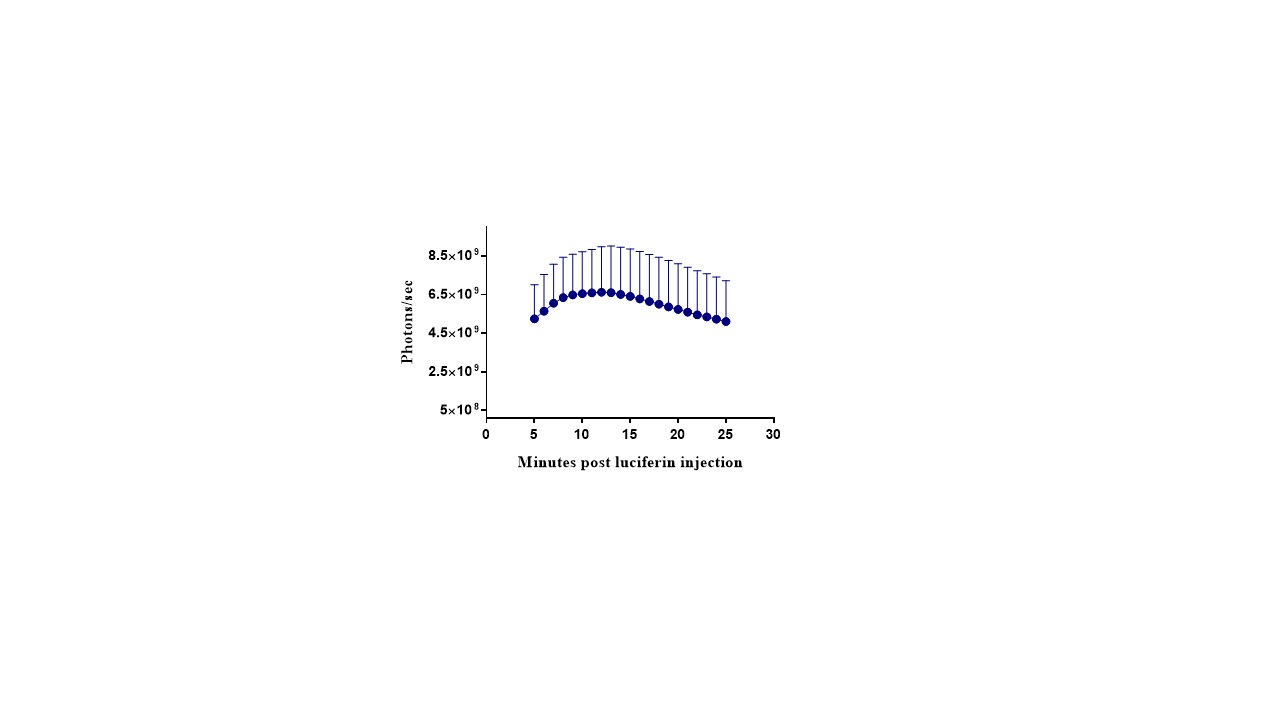

Supplement: Supplementary file 2 — Additional file 2. Luciferin kinetics in ICR-CD1 mice. Luciferin was administered at 200 mg/kg IP to four female ICR-CD1 mice on day 5 post infection with 1 × 10^5^ luciferase-expressing P. berghei infected erythrocytes. Bioluminescence measurements were taken every 1 min starting at 6 min after luciferin administration. Data points represent the mean bioluminescence signal ± SEM for a total of four BALB/c mice for each data point. [file 12936_2019_2661_MOESM2_ESM.jpg]

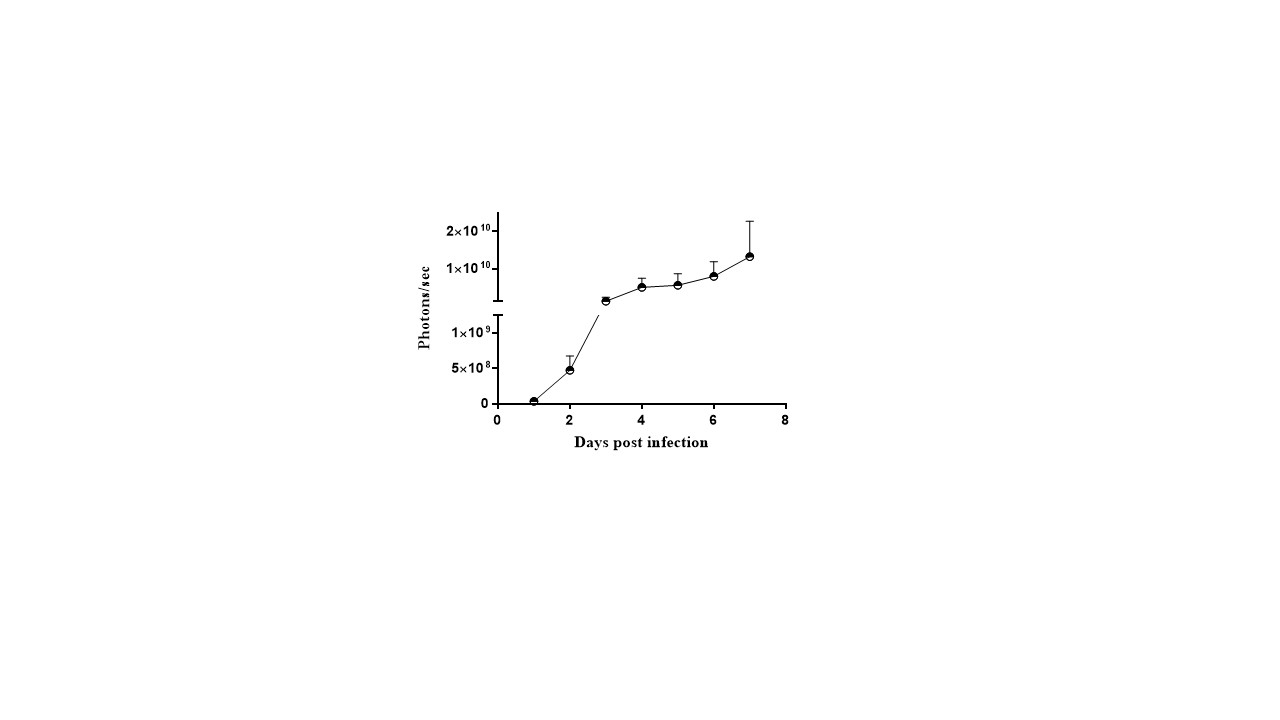

Supplement: Supplementary file 3 — Additional file 3. Evolution of whole-body bioluminescence signal in female ICR-CD1 mice infected with 1 × 10^5^ luciferase-expressing Plasmodium berghei infected erythrocytes. Thirty animals were infected with 1 × 10^5^ luciferase-expressing P. berghei infected erythrocytes on day 0. Whole-body bioluminescence signal was measured on day 1–7 post animal infections respectively on 30, 30, 30, 30, 30, 28, and 24 ICR-CD1 mice, as some animals started being euthanized because of severe malaria on day 6 post infections. The luciferase substrate luciferin was inoculated intraperitoneally (IP) into female ICR-CD1 mice at a concentration of 200 mg/kg, 10 min before bioluminescence analysis. Animals were anaesthetized in a 2.5% isoflurane for 5 min and maintained in the imaging chamber for analysis. Emitted photons were collected by auto acquisition using an IVIS Spectrum instrument. Analysis was performed after defining the animal’s whole-body as a region of interest (ROI). Whole-body total photon emission was quantified using the Living Image software and results were expressed in numbers of photons/s. [file 12936_2019_2661_MOESM3_ESM.jpg]

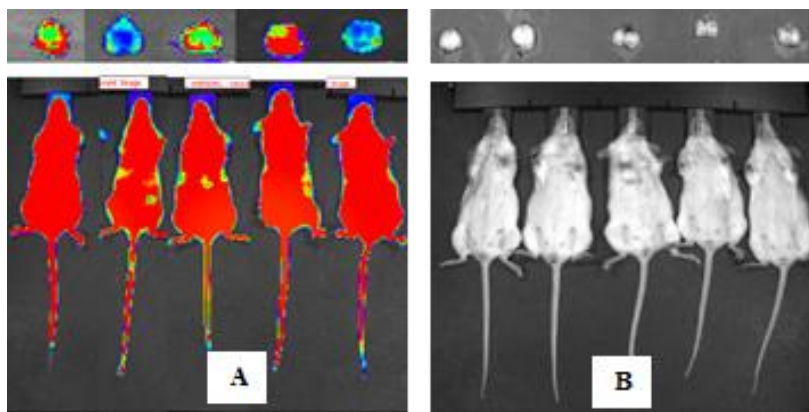

Supplement: Supplementary file 4 — Additional file 4. Luciferase-expressing Plasmodium berghei infected erythrocytes are present in the blood and brain of female ICR-CD1 mice infected with P. berghei parasites. Female ICR-CD1 mice infected with 1 × 10^5^ luciferase-expressing P. berghei infected erythrocytes were injected IP with 200 mg/kg luciferin 10 min before analysis. Whole-body luminescence measurements were taken 10 min post luciferin injections in 10 ICR-CD1 mice. Mice were euthanized immediately thereafter, brains were harvested, and bioluminescence signal was measured. Additional figure 4A shows the bioluminescence signal in the bodies and brains of 5 mice respectively before and after they were euthanized because of severe malaria. Additional figure 4B shows bioluminescence signal in the bodies and brains of 5 mice that belonged to the positive control (mefloquine) treated group respectively before and after they were euthanized on day 31 post infections which is the last day in the study. The photon intensity numbers in the brighter areas of the image (red or bright yellow) are greater than those in the areas with a dimmer green or blue color, where fewer photons were detected. [file 12936_2019_2661_MOESM4_ESM.pdf]

## Slide 1
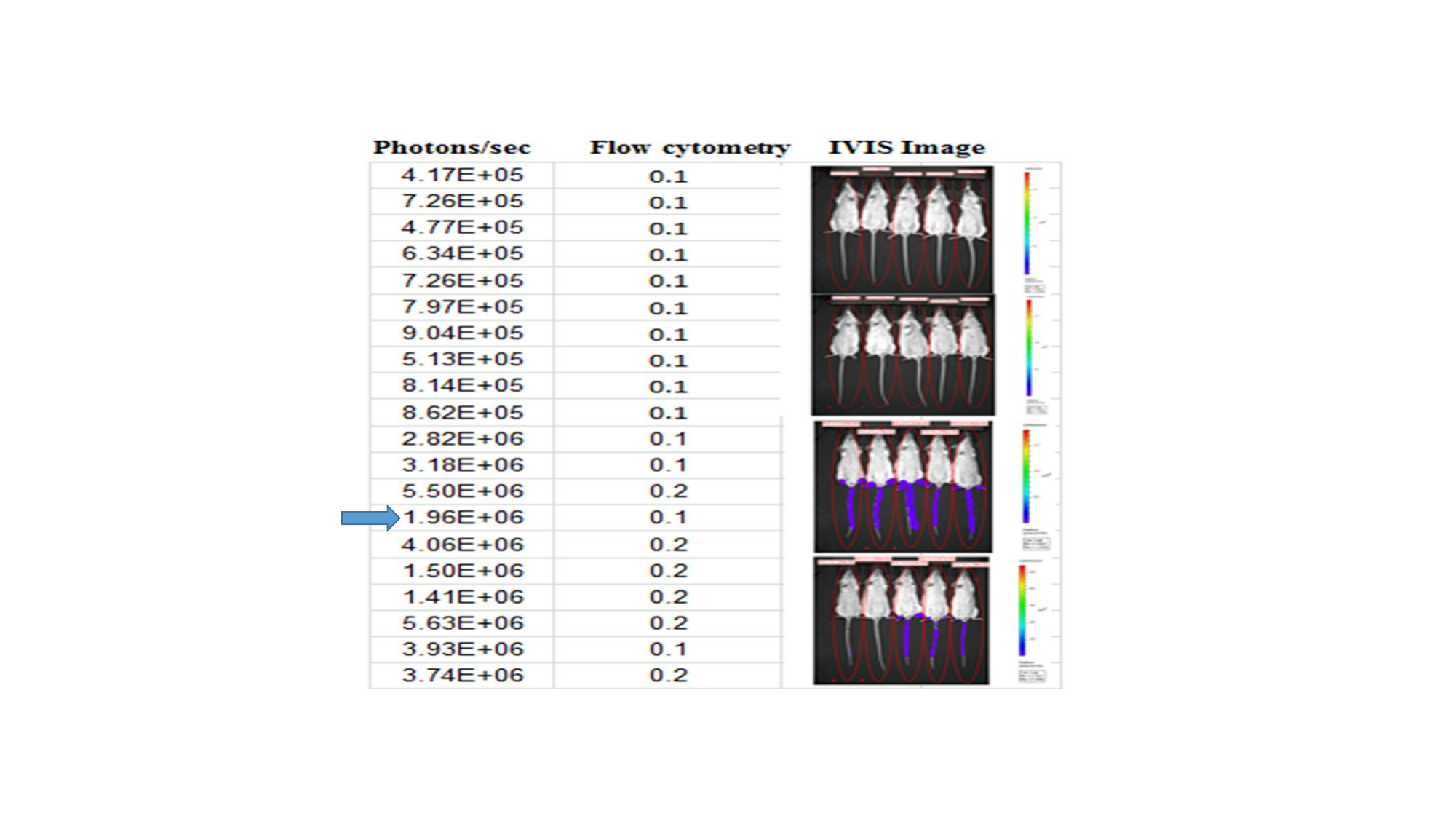

Supplement: Supplementary file 5 — Additional file 5. Limit of detection of the bioluminescence signal in ICR-CD1 study mice infected with luciferase-expressing Plasmodium berghei parasites. Female ICR-CD1 mice infected with 1 × 10^5^ luciferase-expressing P. berghei infected erythrocytes were injected IP with 200 mg/kg luciferin. Whole-body bioluminescence as well as flow cytometry parasitaemia measurements were taken simultaneously. The bioluminescence signal represents the light intensities over the body surface area. Red and bright yellow represent the most intense signal, followed by green and then blue, which represents the weakest signal. The 20 mice showed in this figure had no detectable parasitaemia measured through flow cytometry. Luciferase-expressing P. berghei parasites were visible in the tails of eight ICR-CD1 mice. [file 12936_2019_2661_MOESM5_ESM.pptx]

## Slide 1
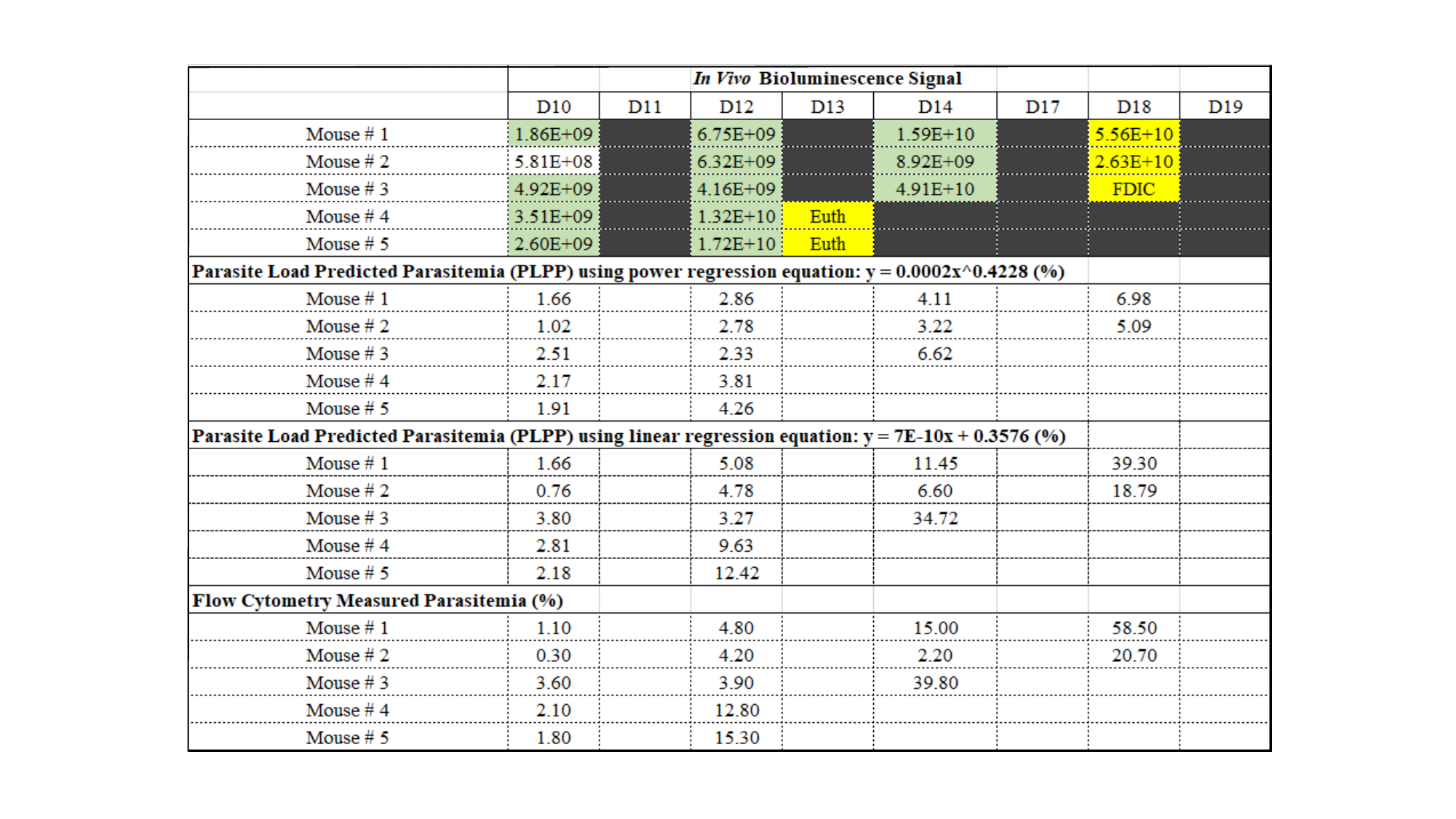

Supplement: Supplementary file 6 — Additional file 6. Use of whole-body bioluminescence signal measurement to animal death in the modified Thompson test studies. Mice 1–5 belonged to a study group which was given 20 mg/kg of a potential antimalarial compound on days 3, 4, and 5 post infections with 1 × 10^5^ P. berghei infected RBC’s. Whole-body bioluminescence values marked in green show the day in which these mice could have been euthanized based in the new criteria for using PLPP to predict animal death in study mice (bioluminescence signal ≥ 1.5 × 10^9^ photons/s). Cells marked in yellow show the day in which these mice were euthanized based on euthanization criteria described in Additional file 1. [file 12936_2019_2661_MOESM6_ESM.pptx]
